# Using Visual Representations to Demonstrate Complexity in Mixed Emotional Development Across Childhood

**DOI:** 10.3389/fpsyg.2021.659346

**Published:** 2021-08-03

**Authors:** Francesca Fotheringham, Matthias Herman, Erin Robbins, Barbara Dritschel

**Affiliations:** ^1^School of Psychology and Neuroscience, University of St Andrews, St Andrews, United Kingdom; ^2^School of Mathematics and Statistics, University of St Andrews, St Andrews, United Kingdom

**Keywords:** mixed emotions, child development, emotional development, analogue emotions scale, protagonist

## Abstract

Previous studies have shown a developmental trend in mixed emotional understanding. As children develop throughout childhood, they begin to recognise simultaneity of positive and negative emotions. However, previous studies have limited ecological validity as they assessed emotion choice using only a single positive and single negative emotion. Therefore, the present study aims to broaden the understanding of mixed emotional development by allowing a wider emotion choice. Mixed emotions were measured using the analogue emotions scale (AES) which allows both intensity of the emotional responses and time to be captured. In the present study, 211 children aged 4–10 were divided into one of three protagonist conditions (self, peer and adult) and read a vignette about the protagonist moving house. Choosing from seven emotions (happy, calm, surprise, sad, worry, fear and anger), they plotted the intensity and duration of each emotion they thought was represented in the vignette. The present study replicated the developmental trend that younger children are more likely than older children to choose a single emotion, and older children are more likely to perceive more simultaneity of emotion than younger children. This trend was demonstrated in the number of emotions chosen, and also the complexity of the AES pattern plotted. Additionally, the present study extended previous research by demonstrating that by broadening the emotion choice, the emotion interaction is more complex than previous studies were able to show.

## Introduction

There are three primary definitions of mixed emotions: the co-activation of multiple emotions, be they opposite or co-valent; the number of emotions experienced throughout a specific time frame in order to fully capture the complexity of the full human experience; or the most common definition, the simultaneous experience of opposite-valence emotions (Charles et al., 2017). Common life events often evoke mixed emotions, such as the death of a loved one after a long battle with illness. Often the bereaved feel saddened by the death, but happy that their loved ones are no longer suffering in pain. Other examples include graduating high school or finalising a divorce, where the individual often feels excited for the future, but nostalgic for the past. For children, the ability to detect mixed emotions has been associated with higher academic performance, more successful social interactions across peer and non-peer situations (Sprung et al., 2015), healthier coping mechanisms, increased creativity, better emotional memory (Gross and Ballif, 1991; Hershfield and Larsen, 2012), greater ability to engage in more sophisticated reasoning (Charles et al., 2017), resilience to stress (Braniecka et al., 2014) and increased empathy (Zajdel et al., 2013).

Given the benefits for children associated with improved understanding of mixed emotions, research has focussed on its developmental trajectory. For example, it has been suggested that children around 5 years old can match multiple emotion face cards to a vignette (Kestenbaum and Gelman, 1995) or can recognise a mixed emotion from an age-appropriate film clip more independently and spontaneously than 3-year olds, who required more support and training (Smith et al., 2015). There is evidence that the ability to recognise a mixed emotion within oneself and others increases in mid-childhood. For example, Harter and Buddin (1987) asked children aged 4–12 years old to describe a situation in which they would feel two emotions simultaneously. Whereas the majority of 4–5-year olds did not describe a situation involving multiple emotions, 6- and 7-year olds reported co-valent emotions directed at either a single target or multiple targets simultaneously, and children older than seven reported opposite valence emotions that were directed to the same target. This finding is consistent with Pons et al.’s (2004) nine component hierarchical model, which posits that comprehension of mixed emotions does not develop until the penultimate component, which occurs around 7–8 years old.

Donaldson and Westerman (1986) also investigated the developmental understanding of two types of mixed emotion pairs, happy and sad, as well as love and anger. They interviewed 4–5-year olds, 7–8-year olds and 10–11-year olds about how someone would feel about getting a new kitten to replace one that ran away and asked which of the emotions from the emotion pairs best described the situation. Consistent with Harter and Buddin (1987), researchers found that the youngest age group usually reported a single positive emotion, while some of the middle age group, and most of the children in the oldest age group reported mixed emotions. This pattern occurred for both the happy and sad condition, and the love and anger condition, suggesting that the effect is not driven by the specific emotions themselves. Larsen et al. (2007) elaborated on this methodology by adding a temporal element that asked children if the chosen multiple emotions were felt simultaneously (at the same time) or sequentially (one after another). Larsen et al. (2007) found that reporting mixed emotions increased with age and that reporting of simultaneous mixed emotions was most prominent in the oldest age group (11–12 years). Larsen et al. (2007) were also able to detect a more nuanced developmental change as they recorded the difference between sequential (one after the other) and simultaneous (at the same time) mixed emotions, whereas Donaldson and Westerman (1986) coded both as mixed emotions. These results highlight that when investigating mixed emotional development time is an important dimension to consider.

Wintre and Vallance (1994) extended previous research on mixed emotional understanding in children by exploring the intensity that the children predicted they would feel for each emotion. In semi-structured interviews, Wintre and Vallance (1994) read children age 4–8-year olds, 15 situations and then asked them to predict what emotion(s), from a selection of five, they would feel and, on a Likert scale, how intensely they felt them. They found that around the age of eight, children demonstrated understanding of opposite valence mixed emotions. They also found that the older children reported more variance in the intensities of simultaneous emotions and concluded that recognising and reporting multiple emotions at varying intensities demonstrates a higher level of emotional comprehension. Therefore, intensity is also a dimension to be carefully considered when exploring mixed emotional development.

It remains an open question the extent to which these data reflect methodological constraints versus developmental differences in how mixed emotions are understood and represented. Many studies of mixed emotion understanding utilise Likert scale ratings, in part, because they were used to validate the circumplex model of emotional processing (Russell, 1980, but see also Russell et al., 1989; Di Blas, 2000; Yik et al., 2000; Yik and Russell, 2003; Tsai et al., 2006; for cross-cultural validations of this work). The circumplex model suggests that all emotions can be represented in a circle around two orthogonal axes, pleasure and activation, with the intersection of these dimensions characterising neutral affect. Emotions separated by 90 degrees are said to be uncorrelated and cannot be experienced simultaneously, whereas emotions separated by 180 degrees are negatively correlated, suggesting that oppositely valanced emotions can only be experienced sequentially (Larsen et al., 2001; Russell and Pratt, 1980). As a result, one limitation of Likert scales as a measure of mixed emotion is that they cannot distinguish between sequential or simultaneous experiences (Carrera and Oceja, 2007).

However, a more recent model of mixed emotions, the evaluative space model (ESM; Cacioppo et al., 1997, 1999) argues distinct neurological activations associated with positive and negative emotions suggest that emotions of opposite valence can be experienced simultaneously (Norris et al., 2010). The model proposes that emotions can be characterised by their relative position in a composite space defined by three dimensions (positivity, negativity and intensity), suggesting that it is possible for discrete emotions to be experienced simultaneously.

The implications from the ESM model led Carrera and Oceja (2007) to develop a methodology that captured both the association and intensity of positive and negative emotions across time. In their method, participants are presented with a perpendicular set of axes, which intersect at zero and where the x-axis denotes time, and the y-axis denotes intensity (the perceived magnitude of the feeling). Participants are asked to plot a line graph of the intensity and duration of happy and/or sad during a vignette. This graph is termed the analogue emotions scale (AES). The choice and intensity of the emotions of the AES plots were validated against 7-point Likert scales of six emotions (happy, calm, surprise, sad, angry and tension).

**Figure 1:**
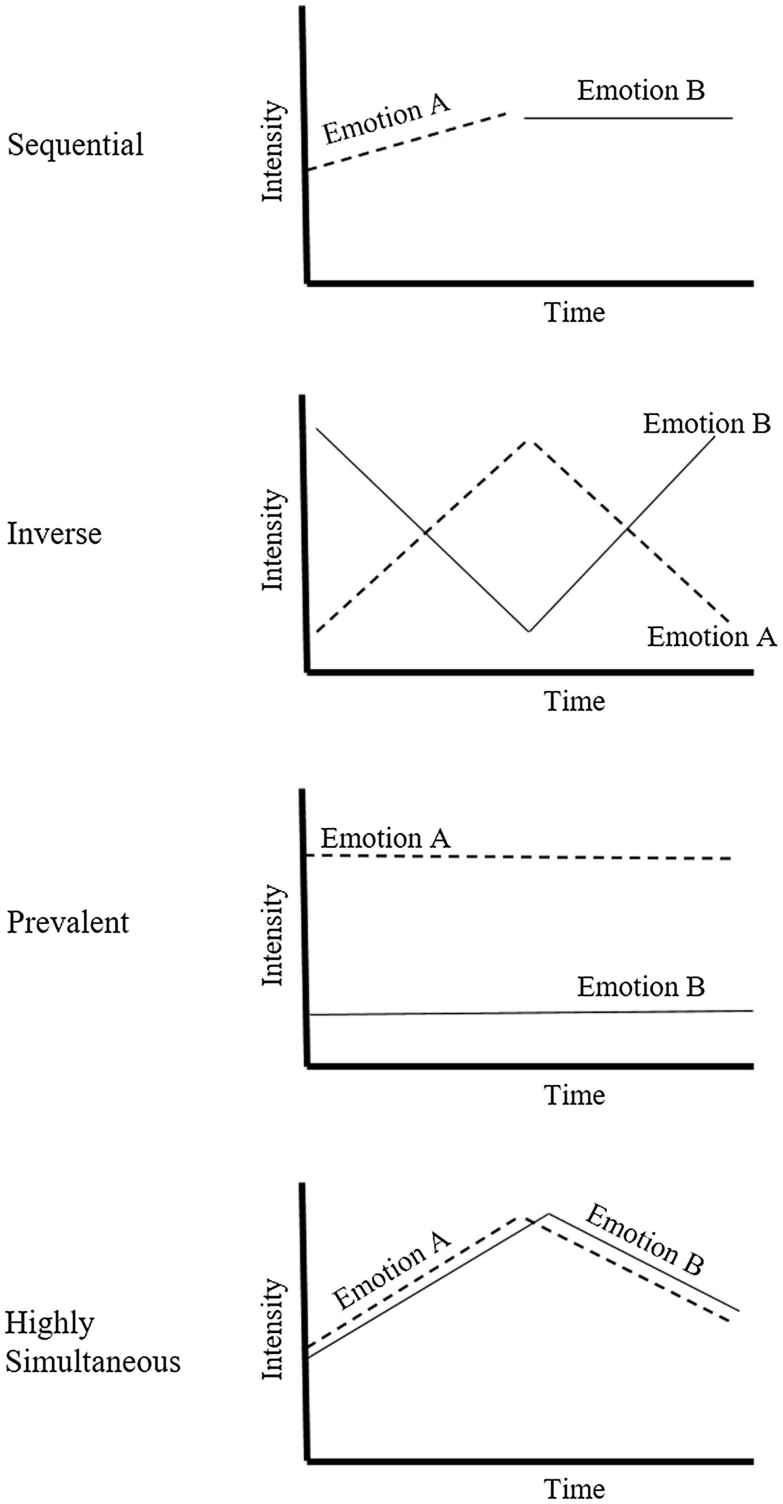
Schematic representation of the analogue emotions scale (AES) patterns as described by Oceja and Carrera (2009).

Carrera and Oceja (2007) and Oceja and Carrera (2009) analysed the AES plots looking for patterns in the intensity and association between positive and negative emotions. Across several replications using varying scenarios (starting university, watching a film clip and reading a fictitious advert) four patterns of mixed emotions emerged as: sequential (no overlapping or crossover of emotion, where emotion B replaces emotion A when it stops); prevalence (both emotions A and B are experienced throughout the time period, yet one has a consistently higher intensity); inverse (as emotion A increases emotion B decreases, and there is a crossover point); and highly simultaneous (both emotion A and B follow the same intensity tract). See Figure 1 for a schematic representation of the four proposed patterns. While the intensity difference between a highly simultaneous AES pattern and a prevalent pattern can inferred from Likert scales, the AES methodology has the advantage of being more sensitive to time as well as intensity.

Burkitt et al. (2018) investigated if the AES could be used to explore how development of mixed emotion understanding occurs in children and hypothesised that the continuum of the AES plots (prevalent, sequential, inverse and highly simultaneous) might change over time. Using an adapted version of the AES, Burkitt et al. (2018) investigated whether children aged 5–7 years represent opposite valence emotions (happy and sad) with different patterns. Participants were read a short vignette where the protagonist (either of themselves or an age and gender match peer) moved to a new house and were asked how the protagonist felt. For children who reported both happy and sad (which was most of the participants), a short interview was undertaken. The purpose of the interview was to validate the AES patterns plotted by the children. The questions were based on the AES plot descriptions and the question order followed the AES continuum: sequential, prevalent, inverse and highly simultaneous. After this interview phase, the children were asked to plot the intensity and duration of their chosen emotion(s) on an adapted AES graph. Using a coding rubric based on the patterns from Oceja and Carrera (2009) and Burkitt et al. (2018) categorised the AES patterns drawn by the participants. They found that there was a high correlation between the AES plots and the interview responses as well as the same patterns as described by Oceja and Carrera (2009) for adults.

A key finding of this study was the observation of a developmental trend, as the younger age group was more likely to report a single emotion and the older age group was more likely to report a highly simultaneous or inverse pattern. This finding was reported for both the AES plots and the interview and across both protagonist conditions. These combined results indicated that the AES can demonstrate a developmental trend of mixed emotion understanding and complexity and also confirms previous findings that children around the age of seven report simultaneity of opposite valence emotions (Donaldson and Westerman, 1986; Harter and Buddin, 1987; Wintre and Vallance, 1994). Therefore, Burkitt et al. (2018) argued that the AES is a valid non-verbal method to research mixed emotions in children. Additionally, they proposed that the patterns accurately show the scope of opposite-valence associations as perceived by children.

Burkitt et al. (2019) further explored the developmental trend of the AES by investigating its applicability in adolescence. Employing a similar method, with participants aged 12–16, Burkitt et al. (2019) explored the AES patterns reported for high arousal emotions (happy/sad) compared to low arousal emotions (relaxed/tired). Contrary to previous evidence (Pons et al., 2004), continuation of mixed emotion development throughout this age group was found. This study demonstrated that mixed emotional understanding is still developing into adolescence as other studies suggested (Donaldson and Westerman, 1986; Harter and Buddin, 1987; Wintre and Vallance, 1994; Larsen et al., 2007). Therefore, these two studies have advanced our understanding of mixed emotional development but leave a gap in the literature as to the AES developmental trend between the ages of 7–12.

Burkitt et al. (2018, 2019) also found developmental differences in reporting mixed emotions for oneself or a peer. While previous studies manipulating the protagonist type found that a child will draw a happy child differently to a happy adult (Burkitt et al., 2011), Burkitt et al. (2018, 2019) found that children more readily recognised mixed emotions in themselves compared to a peer. This finding contradicts other evidence. Larsen et al. (2007) found that children aged 5–12 were more likely to report mixed emotions for the character in the film clip than they were for themselves after watching a short film clip. This result was replicated by Smith et al. (2015) with 3–5-year olds. One explanation for these findings is that emotional expression is inherently social. It is easier to detect portrayed emotional expression of a character (such as vocal intonation and facial expression) than to introspectively detect emotions in oneself. In Larsen et al.’s (2007) and Smith et al.’s (2015) study design, the protagonist did not change perspective across the condition by becoming written in the first person. Deducing how watching a film clip has affected your own emotions, compared to recognising the emotions in the film clip are two separate skills, in which other factors, such as empathy and theory of mind (ToM) play a role. Denham (2005) highlights this point in their hierarchical pyramid of social competence, which integrates Rose-Krasnor’s (1997) model of social competence and Payton et al. (2000) model of social-emotional learning. In Denham’s model emotional self-awareness and social awareness are separate competencies. Results from other methodologies, such as using emotion drawings (Burkitt and Watling, 2015) or semi-structured interviews (Heubeck et al., 2016), also suggest that a recognition of mixed emotions in others develops before a recognition of mixed emotions in oneself.

However, there are some methodological concerns about the findings of Burkitt et al. (2018, 2019). Firstly, the wording of the interview method employed by Burkitt et al. (2018) is considered difficult to comprehend as the questions require a high level of mental manipulation. The questions were also asked in the same hierarchical order, and the interview was only stopped once the child answered ‘yes’, requiring a child to answer ‘no’ six times to an unfamiliar authoritarian figure to report a highly simultaneous pattern. Thus, social desirability in this interview methodology may have given a false or unrepresentative result. Another methodological limitation is that Burkitt et al. (2018, 2019) used the AES patterns as the coding rubric to replicate the AES patterns, with no statistical analysis to ascertain if the patterns were deduced from the data. Therefore, in order to mitigate both these limitations, a data-driven approach giving statistical evidence for the patterns identified is required.

Both Burkitt et al. (2018, 2019) studies restricted the participant to plotting one positive and one negative emotions, raising the question of whether these patterns are representative of real-world emotion associations. Previous research has established that emotions are not experienced as discrete and independent entities (Russell and Fehr, 1994; Saarni, 1999; Posner et al., 2005). It could be considered reductionist to research emotions in this positive and negative emotion dichotomy, when the aims of such research are to explore how a specific cohort thinks emotions interact. To fully explore this question, the number of emotions each participant can plot on an AES graph needs to be widened.

The present study will investigate the development of mixed emotion understanding in children using a more ecologically valid methodology. This will be the first study to analyse the AES using both the previously used coding structure alongside a data-driven approach, within a child population. There has been little research which systematically manipulates the target protagonist in mixed emotion research. All previous research has compared the first person with one other target protagonist type. The present study is the first to investigate the impact of protagonist perspective within three conditions: ‘self’ where the protagonist is in the first person; ‘peer’ in which the protagonist will be an age-matched peer; and ‘adult’ in which the protagonist will be an adult. In line with Burkitt et al. (2018) findings (who compared the ‘self’ and ‘peer’ conditions), we predict that within the younger age group a higher proportion of mixed emotions will be found in the ‘self’ condition as opposed to the peer condition and that the adult condition will have the least reports of mixed emotions. We predict that this trend will also be found in the middle and older age groups. Additionally, it is hypothesised that this trend will also be seen in the number of emotions children in each age group choose.

Another aim is to investigate the impact of greater emotional choice on mixed emotion understanding in order to provide a more ecological test of how children perceive mixed emotions. A limitation of previous research is that only two emotions were used with the AES. In the current study, children will be offered a choice of seven emotions: happy, calm, surprise, sad, worry, fear and anger. The selection is based on the six emotions Carrera and Oceja (2007) and Oceja and Carrera (2009) used in the Likert scales to compare with the AES findings (happy, calm, surprise, sad, angry and tension). As these emotions are closely related to Ekman’s (Ekman et al., 1972) basic six emotions (happy, sad, fear, surprise, anger and disgust), they should be familiar to many young children (Denham and Couchoud, 1990; Weimer et al., 2012). An extra emotion was added to the current study as tension was changed to worry and fear. It was believed children would be more familiar with these emotion words and they also describe two distinct aspects of tension. The children will be trained first how to use an adapted version of the AES scale where the adaptations consisted of making explicit the times periods (beginning, middle and end) where they are asked to identify the emotions experienced and the anchors for the intensity (lots versus little). The children will then be read a vignette and asked to plot the intensity and number of emotions experienced at three different time points in the vignette using the adapted AES.

As the AES methodology has been explored using the definition of mixed emotions being the co-activation of opposite valence emotions, these emotions will initially be split into positive and negative categories and the AES plots will be analysed in terms of these two categories. This will enable the confirmation and replication of the patterns initially found by Burkitt et al. (2018, 2019). The analysis will then be repeated using the full range of emotions to see if similar AES patterns are found using a wider range of emotions. It is predicted that this wider range of emotions will show a more ecologically valid picture of how children think emotions will interact and hypothesise that similar AES patterns will be found.

It is also hypothesised that the AES will be able to demonstrate a developmental trend in reporting mixed emotions. It is hypothesised that the younger age group will be more likely to report a single or sequential mixed emotion pattern, while the older age group will report more highly simultaneous or inverse (when one emotion increases the other decreases) emotion patterns. From the vignette used, we expect to see an increase in positive emotion and a decrease in negative emotion, as detecting this intensity interaction demonstrates a higher-level emotional understanding. We expect this emotion interaction to increase throughout the age groups.

Expanding the number of emotion options will allow for a more comprehensive view of the complexity of emotion associations children believe are being expressed in the vignette. We predict that the number of emotions chosen will demonstrate the recognition of emotional complexity. Therefore, it is hypothesised that the older age group will choose a higher number of emotions, will report opposite-valence emotions at the same time points more often, and plot more highly simultaneous and inverse emotion plots than the middle and younger age groups. Furthermore, as the vignette used in the present study resolves with a positive ending, the expected emotional trajectory, is that negative emotions will decrease across time, and positive emotions will increase across time. It is expected that the oldest age group will detect this expected emotion association more than the other age groups.

In summary, the current study has three hypotheses. First, it is predicted that the AES patterns found in previous research will be identified using both a coding scheme and a data-driven approach. The second prediction is that there will be a developmental trend in the mixed emotion reporting, namely, that the oldest children will demonstrate the most comprehensive understanding of mixed emotions by reporting simultaneous AES patterns more frequently, and choosing the highest number of emotions, as compared to the middle age group who, in turn, will report more simultaneous and inverse patterns and a higher number of emotions than the youngest age group. The youngest age group will report single and sequential AES patterns more frequently than the other age groups and will choose the least number of emotions. Finally, it is predicted that there will also be a difference in the mixed emotion reporting based on the protagonist used in the vignette and this difference will become less prominent with age. Children who hear the vignette with the adult protagonist will report the least amount of mixed emotions, and those who hear the vignette in the first person will report the highest number of mixed emotions. As the children become older we expect that they will be able to take the perspective of others, and therefore, these differences will be reduced.

## Materials and Methods

### Participants

Two hundred and nineteen participants took part in this study. After exclusions, the final sample consisted of 211 participants who ranged in age from 4 years and 0 months to 10 years and 7 months (mean age 7 years 3 months). There were 97 males and 114 females. Seven participants were eliminated due to a parental report of Autism Spectrum Disorder (all male) and one 4-year-old male was excluded as they did not comprehend or concentrate on the tasks. Using an *a priori* power analyses with an alpha = 0.05 and power = 0.4, the projected sample size of 153 would be needed to compare the three age groups by three protagonist groups. Therefore, the sample size of 211 is adequate to complete the requisite analyses. Participants were recruited through the University of St Andrews Baby and Child Laboratory database, and from local science fairs and science centres in the Fife and Dundee area. The majority of participants was recruited from the Fife area which is 94% white Scottish/British (Scotland’s Census, 2011). This study received ethical approval from the University of St Andrews Teaching and Research Ethics Committee.

### Materials

#### British Picture Vocabulary Test Third Edition

This test was used to control for verbal ability. In this test, the child is shown four stimuli pictures simultaneously. The researcher states a word and the child is asked to point to the stimuli picture that corresponds with it. The picture stimuli are grouped in sets of 12, and the test continues until the child gets eight or more incorrect in a set. There are 14 sets in total (Dunn and Dunn, 2009).

#### Adapted Analogue Emotions Scale

**Figure 2:**
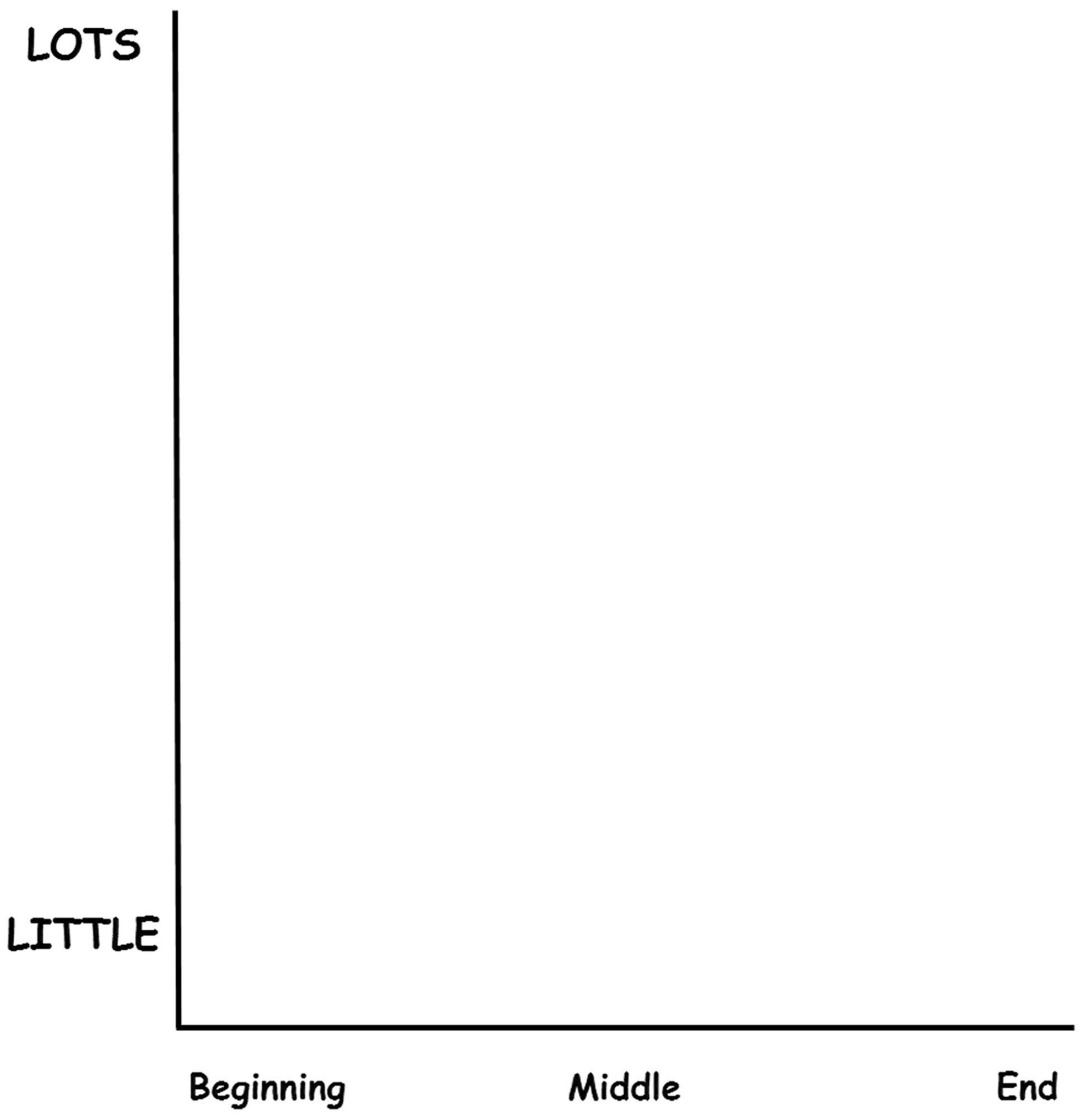
Adapted AES used for the testing phase. The AES has been adapted to include the time point anchors of ‘beginning’, ‘middle’ and ‘end’ along the x-axis, and the intensity anchors of ‘lots’ and ‘little’ along the y-axis.

An adapted version of Oceja and Carrera’s (2009) AES used previously with children by Burkitt et al. (2018, 2019) was employed in both the training phase and the testing phase. The scale consists of a set of two perpendicular axes which intersect at zero. The x-axis denotes the duration of the vignette and is adapted to include three anchoring time points labelled ‘beginning,’ ‘middle’ and ‘end’ equally spaced along the x-axis. Intensity is measured along the y-axis which has been adapted to include two anchors of ‘lots’ at the top of the axis and ‘little’ at the bottom of the axis. The y-axis measures 155 mm on A4 paper. These adaptations were made to facilitate the children’s understanding of how the space within the axes worked. The adapted AES scale (see Figure 2) was printed, and the participants plotted the intensity and duration freehand using Crayola wax crayons (Oceja and Carrera, 2009).

#### Strengths and Difficulties Questionnaire

This questionnaire is completed by parents and consists of two sections. In the first section, the parent completes 25 questions that describe their child’s behaviour. The parents are asked to tick if the child displays this behaviour ‘never,’ ‘sometimes’ or ‘often.’ The second section of the questionnaire asks if they believe their child displays any difficulties, the degree and duration of the difficulties and the impact of these difficulties on the child’s home, classroom, social, leisure activities and family life. While these data were collected, it is not relevant for hypotheses of this paper and is not included in the analyses (Goodman, 1997, 1999).

### Procedure

#### Training Phase

A training phase was conducted initially so that the children could practice graphing intensity and duration. The children were shown an empty AES with the following adapted anchors: ‘lots’ and ‘little’ on the y-axis to denote intensity and ‘breakfast’, ‘lunch’ and ‘dinner’ on the x-axis to denote duration. The researcher explained to the child that they were going to change how many apples were in the bowl at the different mealtimes. The child was instructed to draw lines of how many they thought were in the bowl. The child was told to draw the lines higher up the page if they thought there were more apples and draw the lines lower down the page if they thought that there were fewer apples. Prompts on how to graph the intensity were reduced when the child’s confidence graphing the intensity increased, and the child was observed to be plotting the correct intensity relative to the amount of fruit. The same procedure was followed for the oranges (‘breakfast time’ was one orange; ‘lunch time’ was four oranges and ‘dinner time’ was three oranges). The training phase lasted approximately 5 min.

#### Testing Phase

After the training phase was completed, the child was read a vignette about moving house. The protagonist in the vignette was either in the first person (‘self’ condition), an age-matched peer (peer condition) or an adult (adult condition; see Supplementary Materials for full stories pertaining to each condition). The child was then presented with seven emotions (happy, sad, fear, worry, calm, surprise and anger). Each emotion was presented as a word printed on a card with a corresponding emoji above it. The order for presenting the emotions was randomised by shuffling the cards. The researcher then pointed to the emoji and stated the corresponding emotion when each card was presented. The child was then asked what the protagonist in the vignette felt. The participants were prompted that they could choose as little or as many feelings as they wanted and were reminded that they could hear the vignette as many times as they wanted. Once the child selected their emotions, the researcher read aloud their chosen emotions and confirmed with the child that these were the emotions that they thought the protagonist felt. Next the unchosen emotions were taken away. The participants were then asked to choose a different colour for each of their chosen emotions. Colour choice was limited to pink, red, yellow, orange, green, blue, purple, black and brown, as has been used in other research which explored colour used and emotion (Burkitt and Newell, 2005; Burkitt and Sheppard, 2014). Using one emotion at a time, participants plotted the intensity and duration of each chosen emotion in a similar manner to the training phase. The participants were reminded of each anchoring event in the vignette (first found out about moving, thinking of their friends and first arriving at their new school/job), before they plotted each emotion at each time point. The testing phase was always administered immediately after the training phase. The BPVS was counterbalanced with half completing it before the training phase, and half completing it after the testing phase. The majority of participants also fell within the expected age range of verbal ability according to the British Picture Vocabulary Test (BPVS-3; Dunn and Dunn, 2009), with a strong correlation with the median age within the expected age range (*r* = 0.71).

## Data Analysis

### Scoring the AES

The AES measures the intensity in millimetres of each emotion reported. The x-axis is divided into three time points that correspond to the beginning, middle and end of the vignette. The y-axis reflects the intensity of the reported emotion. The maximum height of the y-axis is 155 mm, which serves as the maximum intensity rating for any emotion plot. When the plotted intensity exceeded the axis space, the maximum intensity was given. The AES pattern was then created by analysing the change in intensity scores across the three time points. To compare our data to previous findings, individual emotions plotted on the AES were averaged into positive emotions and negative emotions. The positive emotions were calm and happy. The negative emotions were sad, worry, fear and angry. Originally, surprise was included as a positive emotion so that the number of positive and negative emotions was balanced. However, surprise was not included in any analyses as it did not correlate with either the positive [*r*(209) = 0.007, *p < 0*.05] or the negative emotions [*r*(209) = −0.106, *p* < 0.05]. Each average positive and negative data point was subsequently plotted for each participant to represent intensity change over the three time points.

A coding guideline for identifying emotional patterns was developed by the lead researcher and two other researchers, based on the graph types described in the previous literature (Carrera and Oceja, 2007; Oceja and Carrera, 2009). The coding employed the following rules. To be coded as a single emotion pattern, only one emotion valence (either positive or negative) should be chosen for the whole AES plot. For a sequential pattern both positive and negative emotions could be chosen, but only one emotion should be represented at each time point. For the prevalent pattern, one emotion must be consistently present at a higher intensity than the other. It was decided that an intensity difference greater than 25 mm between two emotions was the criteria for a prevalent pattern. This criterion reflects at least a 15% difference between the lowest possible intensity and the highest possible intensity. The inverse pattern must have a cross over point demonstrating that as one emotion valence increases the other must decrease. For a pattern to be classified as highly simultaneous, both the positive and negative emotions had to be plotted with a similar intensity trajectory and the intensity between emotion valences had to be less than or equal to 25 mm. To be coded as any of the graph types, the pattern had to be represented at all three time points. If not, or if it fell outside of these descriptions, it was coded in the ‘other’ category. The AES graphs were visually coded using these criteria by the lead researcher and a second coder blind to the hypotheses. Any discrepancies were discussed with the lead researcher and the second coder. Patterns which fell outside of these guidelines were coded as other. Inter-rater reliability was good, Cohen’s Kappa, K = 0.75.

### Consistency of Emotional Choice Within the AES Plots by Age Group

To analyse the relationship between all the emotion options, the data were transposed to give an intensity plot for each emotion at each time point giving 21 (seven emotions × three time points) variables per participant. The data were then divided into the three age groups (4–5 years old, ‘youngest’; 6–7 years old, ‘middle’; and 8–10 years old, ‘oldest’). Non-metric multidimensional scaling (nMDS) was used to analyse the relationship between emotion consistency within the AES plots as the nMDS is more robust when data are non-parametric (Spence and Lewandowsky, 1989). The nMDS is capable of ustilising the Bray-Curtis dissimilarity matrix (in contrast to other dimension reduction statistics, such as principle component analysis (PCA; Legendre and Legendre, 1998). The Bray-Curtis is able to handle large proportions of null responses and will not consider shared absences as being similar. As participants could choose the number of emotions to plot, there was a large amount of null data within this computation. To validate the goodness-of-fit of the fitted nMDS, the widely used STRESS_1_ test was used which gave a value of 0.11 for the youngest group, 0.12 for the middle age group and 0.12 for the oldest age group. Evaluating the data across all age groups yielded a value of 0.08. These results show that the nMDS is an acceptable fit, meaning the plot is statistically significant (Hardle and Simar, 2007; Dugard et al., 2010). Overall, this analysis demonstrates that the children were consistent in how they plotted each emotion.

## Results

### Differences in Mixed Emotion Reporting by Protagonist Type

It was hypothesised that among the youngest age group, there would be more mixed emotion reporting within the ‘self’ condition followed by the ‘peer’ and then ‘adult’ conditions. Contrary to expectations, there was no significant difference between protagonist condition type (self, peer and adult), mixed emotion reporting (single, sequential and mixed emotion) and age group (youngest, 4–5 years; middle, 6–7 years; and oldest, 8–10 years), X^2^(4) = 3.034, *p* = 0.552, 1 − *β* = 0.898. There was also no significant difference between protagonist condition and mixed emotion reported when each age group was analysed separately: youngest, X^2^(4) = 8.513, *p* = 0.074; middle, X^2^(4) = 3.178, *p* = 0.529; and oldest, X^2^(4) = 2.603, *p* = 0.626 (1 − *β* = 0.941). As there were no significant differences involving protagonist type, mixed emotion reporting and age group, we collapsed across protagonist type for subsequent analyses.

### Replication of Developmental Change in the AES Positive and Negative Patterns

As previous assessments of AES patterns only used positive and negative emotions, we first analysed the patterns by collapsing our emotions into two dimensions, positive and negative and using the coding system as described above. It was hypothesised that the present study would replicate the same positive and negative emotion AES patterns reported in the previous literature (Carrera and Oceja, 2007; Oceja and Carrera, 2009; Burkitt et al., 2018, 2019). While we identified the same four patterns of mixed emotions found previously, there was a critical difference in the distribution of the AES patterns across the age groups (see Table 1). In particular, the youngest age group was more likely to plot a single emotion (61%) than the middle (50%) and oldest (40%) age groups. The same was true for the sequential patterns of emotions which were most common in the youngest cohort compared to the middle and oldest groups (11, 1 and 3%, respectively). The opposite was true for the highly simultaneous AES patterns, which were most common in the oldest cohort (5%) and not present in the youngest age group. This effect was more prominent for the inverse AES pattern type, which was most frequently observed in the oldest (33%) and middle age groups (20%) compared to the youngest group (17%).

**Table 1:** The number and percentage of the coded AES patterns when the emotions are averaged into positive and negative emotions.

Age group	Single	Sequential	Prevalent	Inverse	Highly simultaneous	Other
Youngest (*N* = 64)	39 (61%)	7 (11%)	5 (8%)	11 (17%)	0 (0%)	2 (3%)
Middle (*N* = 74)	37 (50%)	1 (1%)	5 (7%)	15 (20%)	2 (3%)	14 (19%)
Oldest (*N* = 73)	29 (40%)	2 (3%)	15 (7%)	24 (33%)	4 (5%)	9 (12%)
Total (*N* = 211)	105 (50%)	10 (5%)	15 (7%)	50 (23%)	6 (3%)	25 (12%)

A significant difference between the AES pattern type plotted and the age group was found [X^2^(10) = 29.84, *p* = 0.001, 1 − *β* = 0.898]. However due to the low frequency of some of the patterns being plotted, there were insufficient numbers to analyse the developmental trend of each AES pattern type. This was due to the majority (62%) of the emotion plots not replicating the AES patterns found in the previous literature (Oceja and Carrera, 2009; Burkitt et al., 2018, 2019).

### Exploration of the Positive and Negative Patterns Using a Data-Driven Approach

**Figure 3:**
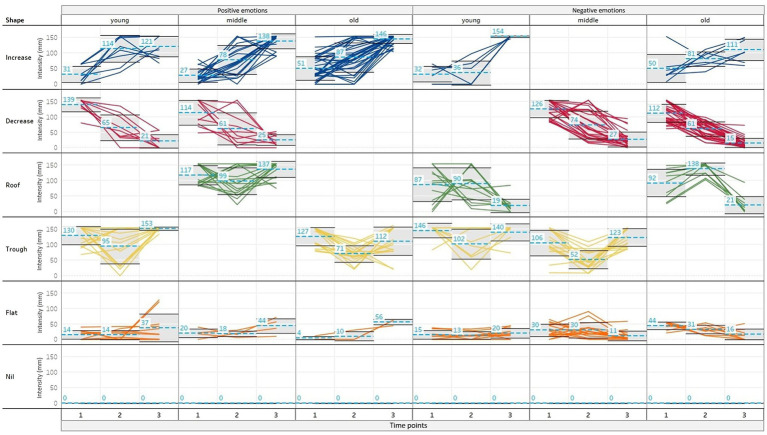
Visualisations of the cluster analyses. The six cluster shapes found are along the y-axis. Within each cluster shape, the emotion intensity (0-155mm) is denoted. The x-axis shows each of the cluster shapes by positive or negative emotion and then by age group (representation created using the software Tableau).

To explore the similarity between observations that are not imposed by any preconceptions of the plot’s pattern, a cluster analysis was computed using R *via* the software Alteryx. A K-means clustering method was employed to ensure that each data point was only in one sub-group, ensuring all the clusters were mutually exclusive. These clusters were computed using the average intensity of the positive and negative emotions plotted for each child for each of the three time points in the vignette. It was decided that four clusters best represented the plotted patterns when adhering to the following requirements: largest difference between clusters (measured by average of cluster and time point; see Figure 3 dotted line); smallest difference within cluster (as measured with standard deviation; see Figure 3 grey box) and having a representative number of participants in each cluster. In each age and valence group, there was one cluster which included all the null intensities and the intensities which had a continuously low intensity. We separated this cluster into two separate clusters so that we could analyse the difference between perceiving an emotion was present a small amount, compared to not perceiving a valence of emotion at all. Overall, six types of cluster shapes were found ‘increase’ (the intensity increasing across the time points), ‘decrease’ (the intensity decreasing across the time points), ‘roof’ (time point one and three have a low intensity, with time point two having the highest intensity) and ‘trough’ (time point one and three have a high intensity, with time point two being the lowest intensity). These patterns were calculated from the K-means time series cluster. Cluster shape ‘nil’ and ‘flat’ were taken from the same cluster. ‘Nil’ was defined as the intensity being at zero at all three time points, reflecting that no positive or no negative emotions were reported. In contrast, ‘flat’ was defined as having a consistently low intensity across all three time points. Within each cluster shape plot in Figure 3, there are three data points, representing the three time points of the vignette. The average intensity of the cluster at each time point is provided in the blue box and blue dotted line. The grey box at each time point shows the standard deviation. The six cluster shapes found are split by age group and by positive or negative emotions. Table 2 shows the percentage, each cluster shape was plotted by either a positive or negative emotion.

It was predicted that this study would replicate the patterns proposed by Oceja and Carrera (2009). Figure 3 and Table 2 show that some findings were replicated. Firstly, the ‘nil’ cluster shape showed that there were many single emotion patterns. Secondly, a highly simultaneous AES pattern was manifested when the same cluster interaction was plotted for both the positive and negative emotions. Additionally, an inverse AES pattern appeared when the positive and negative cluster shapes had opposing intensity. For example, an ‘increase’ cluster shape was plotted against a ‘decrease’ cluster shape, or a ‘trough’ cluster shape was plotted against a ‘roof’ cluster shape. These results suggest a partial replication of the patterns found, as the inverse AES pattern can be accepted by two different cluster shape interactions. However, the cluster analysis approach was advantageous as it revealed more nuanced developmental differences in positive and negative emotion associations.

**Table 2:** The proportion of cluster type reported in the negative emotions for each cluster type reported in the positive emotions.

Positive emotion cluster shapes	Negative emotion cluster shapes						
	Increase	Decrease	Roof	Trough	Flat	Nil	Grand Total
**Youngest age group**							
Increase	0%	0%	8%	2%	2%	6%	18%
Decrease	2%	0%	0%	0%	3%	10%	15%
Roof	0%	0%	0%	0%	0%	0%	0%
Trough	2%	0%	5%	3%	2%	13%	25%
Flat	2%	0%	5%	0%	5%	6%	18%
Nil	5%	0%	5%	8%	6%	2%	26%
Total	10%	0%	23%	13%	18%	37%	100%
**Middle age group**							
Increase	0%	13%	0%	3%	5%	7%	28%
Decrease	0%	4%	0%	3%	4%	3%	13%
Roof	0%	4%	0%	7%	4%	9%	24%
Trough	0%	0%	0%	0%	0%	0%	0%
Flat	0%	1%	0%	0%	3%	1%	5%
Nil	0%	12%	0%	9%	5%	3%	29%
Total	0%	35%	0%	21%	21%	23%	100%
**Oldest age group**							
Increase	3%	27%	4%	0%	5%	11%	50%
Decrease	0%	0%	0%	0%	0%	0%	0%
Roof	0%	0%	0%	0%	0%	0%	0%
Trough	4%	5%	5%	0%	1%	5%	22%
Flat	0%	1%	0%	0%	3%	0%	4%
Nil	3%	14%	4%	0%	1%	3%	24%
Total	9%	47%	14%	0%	11%	19%	100%

*This table is split by age group.*

**Figure 4:**
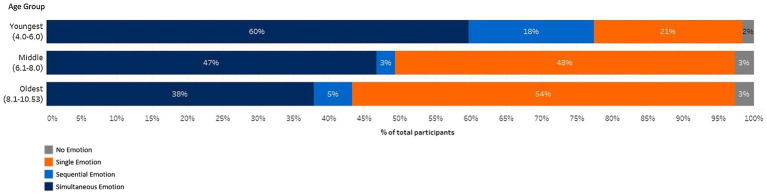
The percentage of children who reported a single emotion, mixed emotions in a sequential pattern or mixed emotions in a simultaneous pattern (representation created using the software Tableau).

As it is believed that children detect more simultaneity in mixed emotions as they develop, it was hypothesised that there would be a higher rate of highly simultaneous and inverse AES patterns reported in the older age group, than the middle and youngest age group. From these six types of intensity trajectories, this would result in four possible cluster shape interactions (positive ‘increase’ with negative ‘decrease’; positive ‘increase’ with negative ‘roof’; positive ‘trough’ with negative ‘decrease’; and positive ‘trough’ with negative ‘roof’). The most dominant of these cluster interactions was positive ‘increase’ and the negative ‘decrease’, which resulted in a clear developmental trend. As can be noted in Table 2, this cluster interaction was reported 0% in the youngest age group, 13% in the middle age group and 27% in the oldest age group. However, if looking at only time point three, where the expectation was that positive emotions would be high and negative emotions would be low, a developmental trend was also found across all the cluster shapes. For the positive emotions, across all the cluster shapes, the frequency of children plotting the expectation of a high intensity at time point three increased from 42% in the youngest age group, and to 72% at the oldest age group. For the negative emotions, 23% of the youngest age group were able to detect that negative emotions should be at a lower intensity at time point three, whereas 35% of the middle age group and 61% of the oldest age group were able to detect this. This suggests a developmental trend of children being able to detect the nuances of the valence structure better as they get older. This was also demonstrated by the ‘nil’ cluster shape decreasing throughout the age groups (youngest 63%, middle 52% and oldest 43%), suggesting that they were reporting both valences more often. The cluster analysis has an advantage over using a coding scheme to identify patterns as it provides detailed insight into the intensity of patterns across all three time points. However, this is at a cost of investigating the sequential and prevalent AES patterns. Some inferences can be made, for finding a sequential pattern of emotion, by delving deeper into the ‘roof’ and ‘trough’ cluster shape interactions. For example, in those interactions where time point one and three in the ‘roof’ shape and time point two in the ‘trough’ shape had an intensity of zero, a sequential pattern could be assumed. However, in the current sample, as only 6% reported this cluster shape interaction, a deeper analysis would not yield accurate results. To examine the sequential pattern further, the original intensities were categorised into the following groups: ‘single emotion,’ ‘sequential mixed emotion’ or ‘simultaneous mixed emotion.’ A single emotion was categorised if only one emotion was chosen and represented at all three time points. A sequential pattern was categorised if only one valence was represented at a minimum of two time points. A simultaneous emotion was categorised if both emotion valences were represented at least two of the time points. This coding was done to explore the stated hypothesis that younger children are more likely to report a single or sequential pattern of emotion. Figure 4 visualises the significant difference [X^2^(6) = 23.19, *p* = 0.001] between age group and level of mixed emotion reported (single, sequential and simultaneous). This result clearly demonstrated that the younger children were more likely to report a single emotion pattern, compared to the older children who were more likely to report a simultaneous emotion pattern.

### Associations of all Possible Emotions by Time Point in Each Age Group

Next, we wanted to examine if the difference in the type of AES plots being reported is due to expanding the choices of emotions. The aim was to ascertain if there was any difference in the AES patterns when all the emotions were taken into account, as compared to when they were collapsed into the two categories of positive and negative. Using the coding scheme adapted from previous researchers, the AES patterns were re-coded to incorporate all the chosen emotions (Table 3). There was a significant difference between the AES coding with all the emotions, and the AES coding when the emotion intensity had been averaged into positive and negative emotions [X^2^(25) = 181.56, *p* < 0.001]. Nonetheless approximately 62% of the AES patterns were still not defined by the previously proposed patterns, with the majority of patterns now being in the ‘other’ category.

**Table 3:** The number and percentage of the coded AES positive and negative interaction patterns when all emotions are included.

Age group	Single	Sequential	Prevalent	Inverse	Highly Simultaneous	Other
Youngest (*N* = 64)	24 (38%)	12 (19%)	2 (3%)	6 (9%)	6 (9%)	14 (22%)
Middle (*N* = 74)	14 (19%)	5 (7%)	5 (7%)	10 (13%)	6 (8%)	34 (46%)
Oldest (*N* = 73)	11 (15%)	2 (3%)	3 (4%)	15 (20%)	8 (11%)	34 (47%)
Total (*N* = 211)	49 (23%)	19 (9%)	10 (5%)	31 (15%)	20 (9%)	82 (39%)

**Figure 5:**
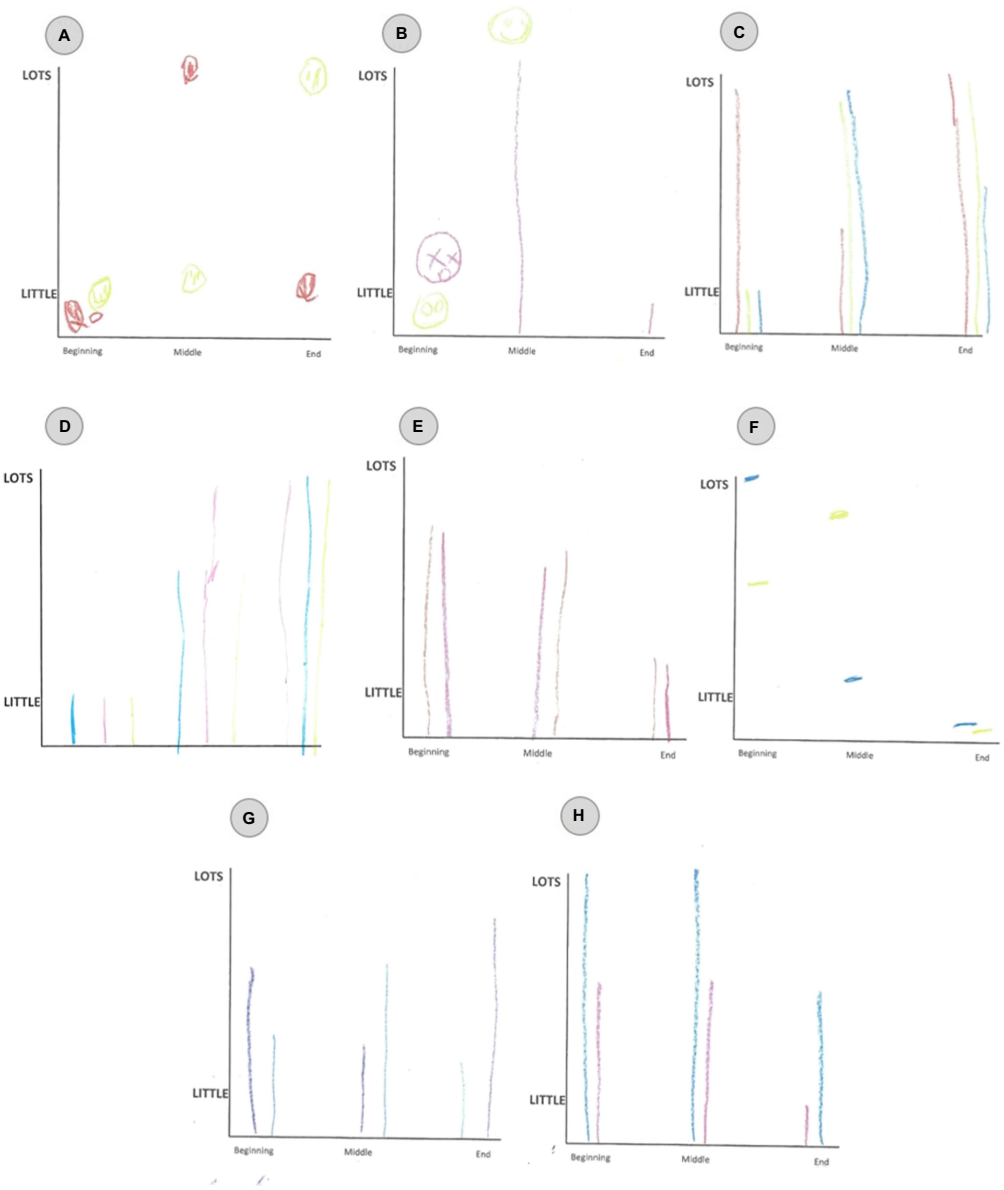
Examples of ‘false single’ emotions AES patterns as drawn by the participants. (A) was produced by a 5 year old male participant. The red colour represents calm and yellow represents happy. (B) was produced by a 5 year old female participant. The pink represents surprise and yellow represents happy. (C) was produced by a 9 year old female participant. The blue represents calm, red represents surprise and yellow represents happy. (D) was produced by an 8 year old male participant. The pink represents worry, blue represents sad, and yellow represents fear. (E) was produced by an 8 year old male participant. The brown represents worry and the pink represents sad. (F) was produced by a 10 year old male participant. The blue represents fear and yellow represents worry. (G) was produced by an 8 year old female participant. The blue represents sad and purple represents calm. (H) was produced by a 7 year old female participant. The pink represents worry and blue represents sad.

There was a significant difference in the number of single emotion plots reported when all the emotions were investigated versus when the emotion intensity was averaged into positive and negative emotions [X^2^(1) = 64.429, *p* < 0.001]. This is because when averaged into only positive and negative, those who reported multiple co-valent emotions were then falsely categorised as single (53%; Figure 5). This shows that when the emotions are averaged into only positive and negative emotions, the richness and variance of intensity between co-valent emotions can be lost. On the other hand, the number of co-valent reporting could be indicative of children not interpreting distinct boundaries between different emotion words. To analyse this further, a nMDS approach was taken to visually represent the relationship between the emotions. The nMDS was used as it is a robust dimension reduction statistic for non-parametric data. For the nMDS, the Bray-Curtis dissimilarity matrix was employed as it is able to handle large amount of missing data. Missing data in this study took the form of null data where children did not choose to plot one of the emotion choices. The Bray-Curtis dissimilarity matrix will not consider this null data as similar. To ensure the model was a good fit for the data, the commonly used STRESS1 test was used. This produced a value of 0.11 for the youngest age group and 0.12 for the middle and oldest age group. When all the age groups were taken together, the STRESS1 test produced the value of 0.08. This value demonstrates that the model fits the data well. This information is difficult to capture using conventional (univariate) statistical methods, but by using this approach, we can demonstrate how the intensity plotted for each emotion at each time point is related to the other time points and other emotions.

**Figure 6:**
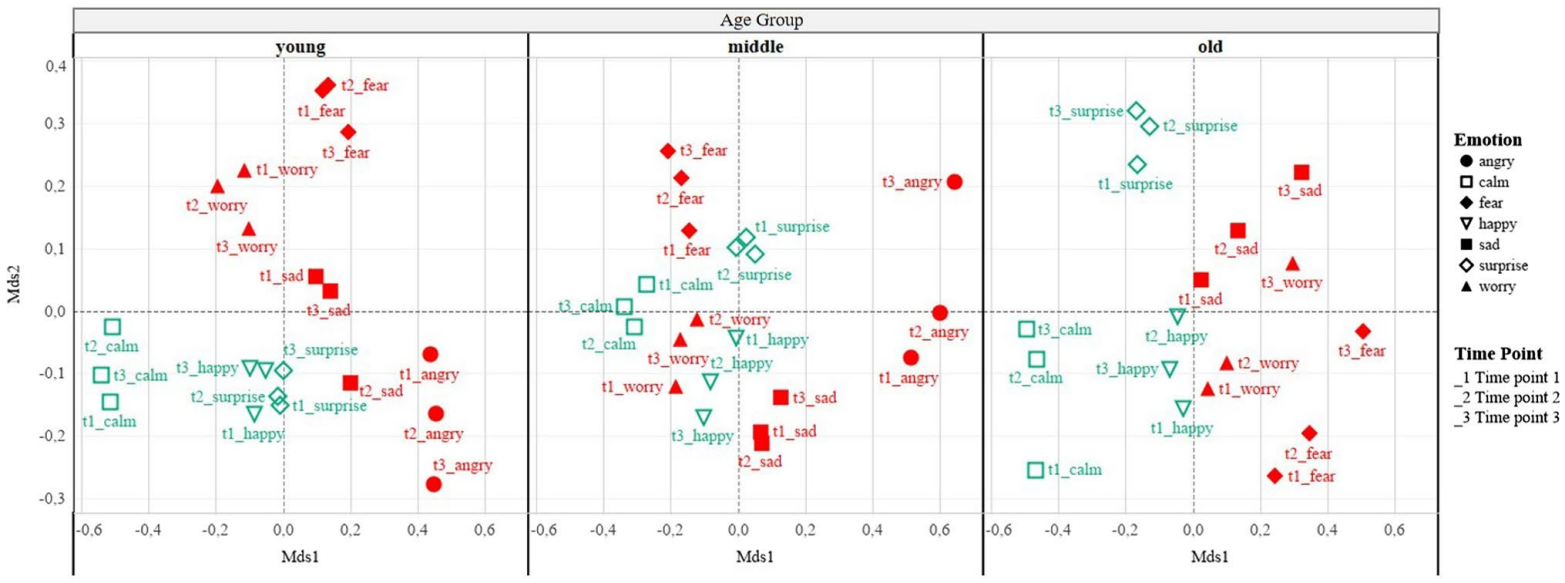
Visualisation of non-metric multidimensional scaling analysis for each age group, where each positive emotion at each time point is shown in green and the negative emotions at each time point is shown in red (representation created using the software Tableau).

Figure 6 shows that the same emotions at all three time-points cluster together, indicating consistency in how the children portrayed the emotions. Interestingly, the oldest age group shows the most consistency in the time point order than the other age groups, with the youngest age group showing the least consistency. For example, the first time point of any emotion is lower than the second time point, and the third time point is the highest of that emotion. ‘Happy’ is the only exception to this pattern. The nMDS also shows that the positive and the negative emotions are more clearly separated for the older age group on the nMDS axis-1, indicating that they have a clearer idea of the valence of the specified emotion. The older children seem to be perceiving the positive and negative emotions to be distinctly different.

The conducted nMDS analysis cannot ascertain if these emotions were plotted as a single emotion pattern, as sequential mixed emotions, or simultaneous mixed emotions. As hypothesised, the older age group are more likely to plot simultaneous emotion patterns (defined as patterns where positive and negative emotions interact across all three time points). We also wanted to explore if the older children were more consistent in their emotion choices and were more likely than the other age groups to plot an emotion choice at all three time points. Figures 3, 6 clearly visualise the developmental trend in reporting the chosen emotions consistently across all three time points as 44% (*z* = −0.42) of the time points in the youngest age contained more than one emotion, compared to 71% (*z* = 0.04) in the middle age group and 81% (*z* = 0.34) in the oldest age group. This suggests that the oldest children are better able to understand the full emotional range of a given situation, rather than being only able to pinpoint the main occurring emotion.

### Number of Emotions

If the older age group reported more simultaneous emotions, it was expected that the number of emotions chosen would also increase. This hypothesis aligns with the definition of mixed emotion being the number of emotions required to fully express the experiences of a given time period. To confirm this hypothesis, a one-way independent ANOVA was computed for the number of emotions chosen by age group, which gave a significant difference [*F*(2, 208) = 10.26, *p* < 0.001, *ƞ*^2^ = 0.09, 1 − *β* = 0.908]. Using Tukey *post-hoc* tests, a significant difference was found between the youngest and middle age group (*p* = 0.017) and the youngest and oldest age group (*p* < 0.001), but that there was no significant difference between the middle and the oldest age group (*p* = 0.163). A one-way ANOVA was also computed for the number of emotions chosen at each time point, which produced similar results (time point one: *F*(2, 208) = 15.57, *p* < 0.001, *ƞ*^2^ = 0.13; time point two: *F*(2, 208) = 14.83, *p* < 0.001, *ƞ*^2^ = 0.13; time point three: *F*(2, 208) = 11.32, *p* < 0.001, *ƞ*^2^ = 0.12). The *post-hoc* tests were also consistent across all three time points with the youngest and middle age groups and the youngest and oldest age groups showing a significant difference. No differences were found between the middle and oldest age group.

To ascertain if there was a significant difference between the number of emotions reported by protagonist type, within each age group, a hierarchical multiple regression was computed. To control for age, age was entered as level 1 and protagonist type entered as level 2. The regression revealed that in the first model, where only age group was included, there was a significant effect on the number of emotions chosen [*F*(1, 209) = 20.24, *p* < 0.001, *R*^2^ = 0.081, 1 − *β* = 0.995] accounting for 8.1% of the expected variance. By adding protagonist type into the regression model (age group by protagonist type), the variation in number of emotions chosen was only increased by 0.3% [*F*(2, 208) = 10.374, *p* < 0.01, *R*^2^ = 0.091]. However, this variance was driven by the age group as in the second model, protagonist type did not produce a significant change to the model (*β* = −0.06, *t* = −0.74, *p* = 0.46) but age group did (*β* = 0.36, *t* = 4.51, *p* < 0.001).

Another hierarchical multiple regression was computed controlling for any differences in the time points. The three time points (beginning, middle and end) were entered as level 1, age group as level 2 and protagonist as level 3. Time point was not significant [*F*(1, 631) = 0.93, *p* = 0.934, *R*^2^ = 0.001] accounting for only 0.1% of the expected variance. When age was included, there was a significant change, as the accounted for 11.7% of the variance [*F*(2, 630) = 34.03, *p* < 0.01, *R*^2^ = 0.118]. In the third level, adding in the protagonist variable only added a 0.2% change in the variance. While the model produced a significant difference [*F*(3, 629) = 23.25, *p* < 0.01, *R*^2^ = 0.12], the coefficients indicate that this was driven by age as this was the only significant coefficient (*β* = 0.41, *t* = 9.14, *p* < 0.001) as time point (*β* = −0.05, *t* = −1.03, *p* = 0.304), and protagonist type (*β* = −0.06, *t* = −1.44, *p* = 0.149) coefficients were not significant.

## Discussion

### The Patterns of Mixed Emotions

The main aim of the current study was to extend our understanding of the developmental trajectory of mixed emotion understanding in children using a visual measure the AES. This was achieved in two ways: by extending the age range used in the previous literature and by expanding the range of emotions in order to increase ecological validity (Burkitt et al., 2018). In the child development literature, this methodology has only previously been investigated with 5–7-year olds (Burkitt et al., 2018) and only two emotional choices. A further aim was to see if a data-driven approach would reveal the same patterns as was obtained with the original qualitative coding scheme. We also investigated if the number of emotions experienced by the protagonist in the vignette would increase with age. Finally, we predicted that with increasing age changes in the types of emotions identified across the three points of the vignette would correspond to changes in the valence of the vignette over time.

Using a similar coding rubric investigating the positive and negative associations found in the AES patterns, a partial replication of the developmental trend identified by Burkitt et al. (2018) was found. Younger children were more likely to plot single or sequential emotion patterns than children in the middle age groups. The appearance of single and sequential AES patterns was further reduced within the older age group who reported more inverse and highly simultaneous emotion patterns. There was no significant difference between the middle and older age groups in the frequency of AES pattern types plotted. These results suggest that the biggest development for children in recognising mixed emotions occurs between the ages of 4–7-years old. This extends the work of Donaldson and Westerman (1986) who demonstrated that there is an increase in mixed emotion recognition between the ages of 4–11 by demonstrating that this increase is not linear, but rather shows a sigmoid function shape.

In the current study, we also investigated the number of emotions reported. We found that there was only a significant difference between the youngest age group and the middle age group. This difference was found both when we averaged across all three time points and when each time point was analysed separately. This finding again suggests that the understanding of mixed emotions often develops most prominently between 4 and 7 years of age. It is consistent with previous evidence which suggests that around the age of 7–8, children begin to recognise mixed emotions, with this understanding increasing as they enter early adolescence (Donaldson and Westerman, 1986; Harter and Buddin, 1987; Larsen et al., 2007). Additionally, they support the Pons et al. (2004) hierarchal model of emotional development, which suggests that mixed emotional understanding occurs around 7–8 years of age.

Although all the patterns reported by Burkitt et al. (2018) were found, there were some key differences. First, there were differences in the proportions of patterns in each category. One of these differences in proportion was that the single emotion reporting was substantially higher across all age groups. The explanation for the higher rate of single emotions being reported than in previous research (Burkitt et al., 2018) might be due to participants plotting co-valent mixed emotions. Thus, the single emotion category reflected the averaging of multiple emotions of the same valence, falsely creating the appearance of a single emotion choice. The results showed that within the positive or negative emotion categories, children detected intensity and duration variances between distinct emotions within those categories. Our results suggest that children have a richer understanding of emotion interaction than previous studies using only a single positive and negative emotion, which also provides further evidence that the definition of mixed emotions should be expanded to include the whole array of emotions, both opposite and co-valent emotions. Previous studies analysing mixed emotional development using only the definition of the coactivation of opposite valence emotions are eliminating this richness of development. Thus, future research should carefully consider using a wider range of emotions including some of the same valence to study mixed emotion understanding. Further by using a wider range of emotional choices, this study found that the positive and negative associations are more complex than previously thought as a significant number of the emotion plots could not be explained by the AES patterns developed by Oceja and Carrera (2009), and subsequently found by Burkitt et al. (2018). They were classified as being in the ‘other’ emotion category. When all the emotions were taken into consideration, this finding was due to the increased complexity of the AES patterns. Children were plotting multiple positive and multiple negative emotions at varying intensities and durations. The findings demonstrate the importance of allowing a wider emotional choice in mixed emotion research. They highlight the need to have a highly sensitive methodology that can detect the dynamic affective choices the participants make over time to ensure the richness of emotional perception is not lost.

In the previous literature (Burkitt et al., 2018), the coding rubric used to classify the AES patterns was based on the four proposed patterns developed by Oceja and Carrera (2009). As the literature using the AES method is still in its infancy, particularly in the developmental literature, a more data-driven approach to ascertain replication of the patterns identified by the coding scheme used by Oceja and Carrera (2009) and Burkitt et al. (2018) is important. The present study addressed this question by examining if a data driven analysis would produce the patterns previously proposed (Oceja and Carrera, 2009; Burkitt et al., 2018, 2019). The cluster analysis yielded six shapes of intensity: ‘nil’, ‘flat’, ‘increase’, ‘decrease’, ‘roof’ and ‘trough’. By using a positive and negative cluster shape together, AES patterns can be found. For example, by using a ‘roof’ shape with a ‘trough’ shape, or an ‘increase’ shape with a ‘decrease’ shape, an inverse AES pattern is found. Similarly, if any of the cluster shapes are the same for the positive and negative emotions, a highly simultaneous AES pattern can be found. These cluster associations, as outlined, were found in the present study. The highly simultaneous patterns observed and the fact that participants reported opposite-valence emotions for the same time points lends support for the ESM model of mixed emotion. This model suggests that positivity and negativity are two separate entities which can be experienced simultaneously regardless of the emotion type (Cacioppo et al., 1997, 1999).

One AES pattern that the cluster analysis did not detect was the prevalent pattern. It would be possible to detect a prevalent pattern using the cluster analysis, if a shape of a consistently high intensity was found in addition to a shape with a consistently low intensity. The present studies cluster analysis only detected a shape with a consistently low intensity. However, the most plausible answer for this is that the present study rarely found the prevalent AES pattern when using the coding rubric (5–7%). Therefore, it was unexpected that this pattern would be detected by the cluster analysis. However, in the previous literature using a similar age group, prevalent patterns were classified at 32% of the sample (Burkitt et al., 2018). There are two possible explanations for this difference. Firstly, this was the first study where children were given an emotion choice wider than one positive and one negative emotion, and therefore, this choice resulted in more nuance and complexity in the mixed emotion patterns. Secondly, in the previous literature (Burkitt et al., 2018), the children were asked questions regarding the AES pattern shape prior to plotting, whereas in the present study, the children were given no indication of possible AES patterns. Therefore, the present study allowed for a more free flowing and thus accurate demonstration of the children’s understanding of mixed emotion interactions. However, this finding requires further investigation to ascertain why this difference occurred.

The cluster analysis also did not detect a sequential pattern, as it analysed the different intensities across all three time points, rather than at each time point individually. However, due to the coding rubric it is known that between 5 and 9% of the sample plotted a sequential pattern (Tables 1 and 3). While this is much lower than the previous literature, it is still a noticeable proportion. An explanation for the difference in the frequency of the sequential pattern might be that in the present study, unlike previous studies, the children were not given any prior knowledge of potential mixed emotion patterns. The sequential pattern displayed also demonstrates the developmental trend of mixed emotional recognition, as this pattern was more frequently plotted in the younger age group than the middle and older age groups. It is thought possible to infer some sequentiality from the cluster analysis. For example, for those participants who plotted a ‘roof’ shape alongside a ‘trough’ shape where the lowest points on both shapes were zero, a sequential pattern could be inferred. However, for this pattern type, it is thought the coding rubric would be the better option, as it can detect small differences between a sequential pattern and a simultaneous pattern with a similar cluster shape which has intensities close to zero. Even though the cluster analysis detected neither the prevalent nor the sequential patterns, there is a trade-off as the cluster analysis provided more insight into the intensity that a coding rubric cannot detect. Therefore, by using the cluster analysis, the present study has been able to gain insights into the intensity changes and interactions in a more precise manner. Thus, the cluster analysis approach may be more sensitive in detecting any differences arising from methodological changes for assessing mixed emotion understanding. The results for the cluster analysis also strengthen support for the ESM model, as they demonstrate that positive and negative emotions are able to be experienced simultaneously.

The use of a wider range of emotions enabled an examination of how the different emotions were related at different time points using a nMDS analysis. This analysis found that across all age groups the time point for each emotion clustered in a group. For example, time point one, two and three for happy were in a similar positioning in the dimensional space. This finding suggests that children are consistent in their understanding and representation of each of the emotions. Further developmental differences in how children detect the chosen emotion to be acting within the vignette were found. Specifically, in the youngest age group, all the emotions at the three time points were grouped together but there was a lack of consistency about the order of the emotions. The order in which the time points appeared within the space became more consistent across the age groups. Namely, in the oldest age group, the emotion at time point one was lowest on the plot, followed by time point two, with time point three for that emotion being highest on the graph. This pattern was the same for all the emotions except happy. These findings again suggest that as the children get older, their understanding of how emotions function within the vignette becomes more consistent. However, it is beyond the scope of the analysis to ascertain how the emotions were interpreted. Both these findings (the clustering of the emotions and the ordering of the time points for each emotion) from the nMDS suggest that the older children have a better understanding of emotions and how they are likely to be felt in a given vignette. They are consistent with the previous literature which shows that throughout this age period, children are able to better recognise happiness, sadness, fear, surprise and disgust (Denham and Couchoud, 1990; Weimer et al., 2012). However, by using the AES and the nMDS analysis, the present study is able to extend the previous literature as it demonstrates how children believe emotions interreact with each other (Donaldson and Westerman, 1986; Harter and Buddin, 1987; Wintre and Vallance, 1994; Kestenbaum and Gelman, 1995; Larsen et al., 2007; Smith et al., 2015).

The nMDS analysis also demonstrated that there appears to be an age effect on the ability to distinguish between positive and negative emotions. The grouping of the positive and negative emotions becomes more distinct with age. The oldest age group showed the clearest separation, suggesting that they understood positive and negative emotions to be separate entities. Previous research (Donaldson and Westerman, 1986; Larsen et al., 2007; Smith et al., 2015; Burkitt et al., 2018, 2019) using only a single positive and negative emotion was unable to investigate this issue. By using a wider emotion choice, this study indicates that children also have a better ability to distinguish between positivity and negativity with age. This finding also supports the ESM model (Cacioppo et al., 1999) which suggests that positive and negative emotions are separate biological entities. It would be interesting to investigate if there is a correlation between the biological development of these separate substrates (bottom-up approach) and the psychological recognition (top-down approach) of mixed emotions (Lindquist, 2013) to investigate any individual differences in AES patterns and how children recognise the relationship between multiple emotions.

In summary, when we employed a wider range of emotions to investigate the understanding of mixed emotions, more unclassified patterns arose when the coding scheme developed by Oceja and Carrera (2009) was employed as compared to previous research findings (Burkitt et al., 2018, 2019). A data-driven approach was also applied to see how the data were clustered into patterns. The cluster analysis revealed that only two of the proposed AES patterns were found (highly simultaneous and inverse; Oceja and Carrera, 2009). Moreover, there was evidence of ‘false single’ emotion patterns where it became apparent that multiple emotions of the same valence were originally selected but had to be averaged when comparing positive and negative emotions. Therefore, we conclude that these findings indicate that using a wider emotion choice demonstrates more and new complexity to children’s recognition of multiple emotions, than previous developmental work using the AES allowed. Additionally, the nMDS is an important analysis for confirming the interaction of the mixed emotion plots as it highlights that there is a lot more to explore in mixed emotional understanding research than simply comparing the intersection of positive and negative emotion. For example, the nMDS demonstrates that children’s consistency in using the emotions in the present study increases across childhood, and that they differentiate between positive and negative emotions more clearly in the older age group.

### Limitations of the Current Work and Future Directions

One limitation of the study is that the number of positive and negative emotion choices was not balanced for our analyses. We had to drop surprise as it did not correlate with the other positive emotions. This reduction in one positive emotion may have affected why ‘happy’ was chosen far more frequently than any other emotion. Another reason was that happiness was the only high arousal positive emotion as calm was included as a positive emotion but is low in arousal and closer to neutral. It could also be argued that happiness was an emotion that the children were familiar with (as it often develops first; Denham and Couchoud, 1990) or that happiness was widely recognised within the vignette. However, sadness is also an emotion commonly recognised by younger children, yet it was not chosen more frequently. Future research using this methodology should change the proportion of emotions used so that they are balanced. Another high arousal positive emotion should be added to ascertain if children were recognising happiness or simply positivity. Additionally, fear and worry could be replaced by scared which would balance the number of positive and negative emotions. It was reported that ‘scared’ is an emotion word used by health and wellbeing teachers in Scottish school curriculum, and therefore, this would be an emotion the children would be more familiar with. In addition to this, it has been reported that worry is often not recognised until later in childhood (Harris et al., 1987; Widen and Russell, 2008). As it could not be determined if surprise was a positive or negative emotion it should not be employed in further studies examining the AES patterns in terms of positivity and negativity. We understand that this is due to surprise being a believe based emotion, whereas the other choices were all knowledge-based emotions (Weimer et al., 2012). It would be interesting to see in future research the affects emotion word choice has on mixed emotion reporting when children use an AES.

While the work using the AES has been able to demonstrate a deeper understanding of mixed opposite-valence emotional experiences, future studies could explore if children perceive co-valent emotion words as similar or discrete. Such research could further our understanding of how children believe positive and negative emotion factors function. Additionally, by further investigating the use of emotion words, one could ascertain if children are using one emotion word to signify a unitary feeling, or if they are using multiple words to try and explain a feeling that they do not yet know the word for. One example would be if children use fear and worry to signify nervousness. Additionally, it would be interesting to investigate the use of more complex emotion words on the AES, such as surprise, grief and bittersweet, which refer to a linguistically singular emotion, yet reflect both positivity and negativity.

A key aspect in using this methodology is that the children have a firm understanding of intensity and duration of emotion. We feel that due to the training phase, children were able to demonstrate that they grasped or understood the mentalising of the two-dimensional space in order to represent the emotions. Therefore, those children who did not demonstrate this understanding during the training phase or who struggled to concentrate on the task for the full 20 min were excluded from the study. As a result, it could be argued that there is an aspect of systematic bias in using this method, and that the excluded participants relied on a vastly different representational schema to represent emotional intensity and duration. Therefore, future research should compare other methods of measuring intensity of multiple emotions, such as volumetrically or using dials. Such methodologies could also be applied to cross-cultural research where graphical representations are not as prevalent.

This study did not find any protagonist effects which are in contrast to findings from the previous literature (Burkitt et al., 2018, 2019). This lack of replication could be due to the high level of cognitive load in the present study. As the child was required to remember the vignette, all the various chosen emotions, and the new skill of using the AES simultaneously, additionally thinking about how a different type of protagonist would have experienced the situation would have greatly increased the cognitive load. Further the task instructions placed greater emphasis on thinking about the various emotions as opposed to the protagonist which was only mentioned at the beginning of the vignette. This emphasis could also explain why protagonist type was ignored. Furthermore, the testing often took place in science museums, or science festivals, where there were other distractions and noises present throughout the testing period. Even though these effects were mitigated as much as possible by testing in a quiet area, they could have still impaired the children’s concentration. In contrast, it may be that children do not differentiate between protagonist type when determining the experienced emotions in a story, as the previous research investigating protagonist effects in children’s understanding of mixed emotions using the AES, only found an interaction of protagonist type (self and peer), for the prevalent AES pattern and protagonist type did not produce any main effects. However, when using other methodologies for reporting mixed emotions, such as Likert scales, these effects were stronger (Larsen et al., 2007; Burkitt et al., 2011; Smith et al., 2015). Therefore, future research is required to understand the difference protagonist type has when plotting emotions versus reporting emotions using other methods. To advance our understanding of the development of mixed emotion understanding, there needs to be research on the cognitive mechanisms that underpin the mixed emotion reporting using the AES. One possible mechanism is ToM. ToM is a cognitive mechanism which develops throughout childhood and requires an individual to understand the connection between a targets emotional state and the social situation to predict behaviour (Wellman, 2014). ToM abilities have been shown to correlate with emotional understanding (Hughes and Dunn, 1998; Cutting and Dunn, 1999; Dunn, 2000; Harwood and Farrar, 2006; O’Brien et al., 2011; Ornaghi et al., 2016; Grazzani et al., 2018). As one aspect of ToM is the ability to take the perspective of another, it could provide further insight into the relationship between protagonist type and mixed emotional understanding. It may also provide new understanding about why mixed emotion reporting increases with age.

### Conclusion

This study expands the previous literature investigating the development of mixed emotion understanding in children using a wider range of emotions than the simple positive and negative dichotomy using the AES method. As previous studies found a developmental trend in children aged 5–7 years old (Burkitt et al., 2018) and 12–16 years old (Burkitt et al., 2019), the present study extended the age gap within the younger age range. The present study demonstrates that recognising a simultaneous association between positive and negative emotions increases throughout childhood. This study was also able to show for the first time, through a data-driven approach the frequency and developmental trend of more subtle positive and negative associations, and the sensitivity of these associations when there is an emotion choice wider than two. In addition, the wider emotion choice also demonstrated that as children develop, they are able to detect a higher number of emotions and understand their complex association throughout a given vignette. Therefore, it would be interesting to see future research continue to explore this trend by using multiple positive and negative emotions and using a data-driven approach at various age ranges so that a deeper understanding of the developmental trajectory can be explored.

## Data Availability Statement

The raw data supporting the conclusions of this article will be made available by the authors, without undue reservation.

## Ethics Statement

The studies involving human participants were reviewed and approved by the School of Psychology & Neuroscience Ethics Committee at the University of St Andrews. Written informed consent to participate in this study was provided by the participants’ legal guardian/next of kin.

## Author Contributions

FF, ER and BD designed the study. FF recruited participants and collected the data for the study. FF and MH performed the statistical analysis. FF wrote the manuscript with MH contributing to the results section. BD contributed to other sections of the manuscript. All authors contributed to revisions and approved the submitted version of the manuscript.

## Conflict of Interest

The authors declare that the research was conducted in the absence of any commercial or financial relationships that could be construed as a potential conflict of interest.

## Publisher’s Note

All claims expressed in this article are solely those of the authors and do not necessarily represent those of their affiliated organizations, or those of the publisher, the editors and the reviewers. Any product that may be evaluated in this article, or claim that may be made by its manufacturer, is not guaranteed or endorsed by the publisher.
